# The Importance of Edible Films and Coatings for Sustainable Food Development

**DOI:** 10.3390/foods11203221

**Published:** 2022-10-15

**Authors:** Carmine Summo, Davide De Angelis

**Affiliations:** Unit of Food Science and Technology, Department of Soil, Plant and Food Science (DISSPA), University of Bari “Aldo Moro”, Via Amendola, 165/A, 70126 Bari, Italy

Nowadays, it is crucial to adopt an integrated approach to improve the sustainability of the food system that covers the whole supply chain, from primary production to packaging and distribution. Indeed, packaging has a considerable impact on the environment, and it is estimated that it contributes to about 35% of the municipal waste produced in the European Union as a whole, with waste production increasing annually [1,2]. Packaging in the food sector has the primary role of preserving food quality and ensuring that food products are safe from (i) physical damage, i.e., protection against light and mechanical damage, and guaranteeing the desired permeability to gases and solutes and (ii) microbial spoilage or chemical modifications, therefore extending the shelf life of products [3,4]. The design of food packaging should be adapted to the intrinsic and extrinsic characteristics of the product itself in order to assert the above-mentioned functions [3]. Moreover, as it is utilized at the end of the supply chain, it is crucial to develop and apply efficient packaging systems that can minimize food loss and waste. Indeed, it is estimated that food loss through the whole supply chain accounts for 30% of the waste in total food production [1]; therefore, losses due to poor packaging have a strong impact on the environment.

From this perspective, the development of edible films and coatings could be an environmentally friendly response to packaging waste. An edible film or coating is defined as “*any type of material used for enrobing (i.e., coating or wrapping) various foods to extend shelf life of the product that may be eaten together with food with or without further removal*” [5]. In particular, films are stand-alone materials that adhere to the food and that may or may not be removed before consumption, whereas coatings are formed directly on the food and are consumed together with it [4,5,6]. Both generally have a thickness of less than 0.3 mm [5].

It may be surprising to learn that the edible films and coatings have a long-established history of utilization in vegetable and meat products as preservers [5,7], and, as it often happens, traditional technologies can be rediscovered and readapted to the present day from the perspective of promoting sustainability. Certainly, several efforts are needed to develop greener and more sustainable packaging systems [3].

The current state of the art regarding the use of edible films and coatings in the food sector has recently been reviewed by several authors [4,5,6,7,8]. In particular Díaz-Montes and Castro-Muñoz [4] comprehensively reported on the main aspects related to edible films and coatings such as biopolymers and the additives, requirements, and properties of such materials as well as on the manufacturing methods and the applications of these materials, focusing on the recent research carried out on several categories of food products. For example, Panchal et al. [6] provided deep insight into edible films and coatings as they are applied to fruits and vegetable, whereas Umaraw and Verma [8] reviewed the application of edible films on meat and meat products. In these reviews, the important aspects related to the laws and the international regulations for food packaging have also been reported [4,6].

It is important to highlight the versatility of the edible films and coatings being produced using different materials and that have been adapted to a wide range of food products. In particular, the possibility of producing edible films and coating using side streams and the by-products generated by the food supply chain has been widely investigated, pointing out that the implementation of circular economy strategies is achievable for food-packaging materials. For example, edible films have been developed using the proteins and the pectins generated from the pumpkin industry [9], orange peel extract [10], mushroom by-products [11], cheese whey and orange peels [12], and, overall, the waste produced by fruit-processing operations [13].

Furthermore, researchers have addressed efforts to ensure that packaging is not only edible, but also active through the inclusion of bioactive molecules [4,14] and/or microorganisms such as lactic acid bacteria or fungi [4,15]. Active packaging is a system in which the product, the environment, and the packaging interact to produce a beneficial effect, e.g., prolonging the shelf life of products [16]. Packaging manufactured with bioactive molecules such as phenolic compounds has the advantage of boosting protective activity against the oxidation phenomena that may occur during storage [4]. Moreover, essential oils, organic acids, enzymes, and peptides have been demonstrated to have an inhibitory effect against pathogenic bacteria and antifungal activity [4].

Innovative strategies under development for edible films and coatings involve the entrapment of probiotics, as previously reviewed [4,15,16]. The incorporation of probiotics in packaging could support the optimal viability of the beneficial microorganisms in food, helping to guarantee the healthy effect of the food on consumers [15], and paves the way for the development of a wide range of food solutions in which the packaging and the food can have a synergistic effect.

Edible films and coatings should have mechanical and barrier properties comparable to those of conventional packaging materials so that they can be used in the food industry with ease. A recent review evaluated the state of the art regarding the application of non-thermal treatments and nanocomposite materials to improve the properties of edible films, highlighting that additional research is needed to reduce the costs of these materials before their widespread utilization can be achieved [17].

In conclusion, edible films and coatings have strong potential to aid sustainable food production by reducing packaging waste, extending the shelf-life of the products, and actively interacting to preserve food quality. Moreover, the possibility of being integrated with circular economy strategies makes edible films and coatings versatile and capable of reducing the environmental impact of food systems. Overall, further efforts are needed to scale-up the developed technologies at the industrial level and to support the diffusion of edible films and coatings.

## Data Availability

Not applicable.
